# The Antiseptic Treatment of Gonorrhea

**Published:** 1894-08

**Authors:** Edward Pendleton

**Affiliations:** Owensboro, Kentucky


THE ANTISEPTIC TREATMENT OF GONORRHEA.—Gonorrhea is the most common venereal disease with which we have to deal. That it can be acquired late in life is shown by Dr. G. E. Isaacs, of New York, who reports a case in the *Medical Record*, April 14, 1894, occurring in a man aged one hundred and three years. When one hundred years old he contracted chancroids, and at one hundred and three had an attack of gonorrhea! Many cases are recorded of infants and children having gonorrhea. The first attack is usually the severest.

Gonorrhea is caused by some mucous membrane, usually that of the urethra, coming in contact with some septic poisonous matter, generally derived from one of the opposite sex. Gonorrhea is highly contagious and infectious. It is a self-limited disease; cases are on record in which no treatment was employed with gonorrhea, and it has been found to get well after it had run its course, which may be from three to six weeks. The period of incubation after exposure to gonorrhea is from a few hours to a fortnight. The early symptoms are a red and swollen meatus, slight burning sensation when urinating, the mucous discharge characteristic of the disease is soon noticed, at first especially in the morning.

The complications are balanitis, paraphimosis, prostatitis, cystitis, epididymitis, orchitis, bubo, ophthalmia, and retention of urine.

The diagnosis of gonorrhea is not always easy, but, as a rule, a physician can make the diagnosis inside of six days. Where there is any doubt at the end of this time a microscopical examination of the discharge should be made. If gonococci are present the diagnosis is certain. It is always well, if possible, to examine the genital organs and discharge of a patient presenting himself for the treatment of gonorrhea.

The treatment in the acute stage should be the following: Rest in bed for six days. Drink no alcoholic stimulants or coffee; eat as little meat and salty substances as possible; keep the bowels well open by eating fruit daily. Abstain from all sexual excitement, as this produces chordee, which is a very disagreeable complication. The patient should take a cold bath each morning; this will not only refresh him but will keep him clean. The testes should be suspended in a suspensory bandage, to guard against orchitis.

It is the duty of every physician treating a case of gonorrhea to instruct his patient how to use the syringe. The patient before using his injection should urinate, then wash the penis, especially the glans, in warm carbolic acid solution (one to thirty) followed by an injection of simple warm water into the urethra, so as to wash away the pus and render the parts aseptic. Cases are recorded in which patients suffering from gonorrhea, instead of injecting the solution in the urethra, have injected it under the prepuce. The patient should lie in the recumbent position, previously having washed his hands in a warm carbolic solution. The two drachms of the injection to be used should be poured into a clean cup; after shaking the bottle, a syringe with a blunt point should next be annointed with vaseline and the solution drawn up into the syringe. The patient should be taught how to introduce the syringe into the meatus, as a stricture is often produced at the anterior portion of the urethra, due to rough manipulation and an unsteady hand. He should inject the fluid slowly, not forcibly; quite often a posterior urethritis is produced by forcing the pus back of the compressor urethræ muscle. The fluid should be held in the urethra from one to three minutes, when it is allowed to run out. The patient should have a sock with absorbent cotton loosely placed in the toe, so suspended as to catch all of the discharge. It is never well to cover the glans penis with cotton,—in this way the acrid discharge is retained in the urethra; by using the sock the soiled cotton can be changed from time to time when necessity demands. Two strips can be attached to the sock and fastened to the suspensory belt which passes around the waist.

After gonorrhea has persisted for six days the following injection may be used with advantage:

| | | |
|---|---|---|
| R. | Acid. borici ........................................................ | ʒii. |
| | Tinct. iodi ........................................................ | ʒ ii. |
| | Glycerini ........................................................ | ℥ i. |
| | Aquæ q. s. ad ........................................................ | ℥ iv. |
| M. | Sig.—Inject three times daily. | |

If great pain on micturition is complained of, a decoction of red saunders and saw palmetto may be given, a teaspoonful four times daily, so as to dilute the urine and render it alkaline. The patient should wash over and around the glans penis night and morning with a carbolic solution one to thirty, followed by cleansing and disinfecting the hands; by so doing be will be unlikely to inoculate any other mucous membrane.

After there has been no discharge of pus for ten days, the patient may be dismissed as well, with the caution that he must be very careful to avoid sexual excitement and dietetic errors.—*Edward Pendleton, M.D., Owensboro, Kentucky, in Inter. Med. Mag.*

